# Supplementary material for: Heparin-based hydrogel scaffolding alters the transcriptomic profile and increases the chemoresistance of MDA-MB-231 triple-negative breast cancer cells
Source: Biomater Sci. 2020 Feb 13;8(10):2786–96. doi: 10.1039/c9bm01481k (PMC7497406; doi:10.1039/c9bm01481k)
Supplement: Supplementary file 2 [file BM-008-C9BM01481K-s002.zip › Supplementary File 4/HP-BhydrogelvControl/Pathways/my_analysis.Gsea.1545201079907/HALLMARK_COMPLEMENT.html]

Details for gene set HALLMARK\_COMPLEMENT[GSEA]

|  || Dataset | expr.class.cls#HYD\_versus\_CONTROL.class.cls#HYD\_versus\_CONTROL\_repos |
| Phenotype | class.cls#HYD\_versus\_CONTROL\_repos |
| Upregulated in class | CONTROL |
| GeneSet | HALLMARK\_COMPLEMENT |
| Enrichment Score (ES) | -0.35981685 |
| Normalized Enrichment Score (NES) | -1.6400043 |
| Nominal p-value | 0.0 |
| FDR q-value | 0.007217505 |
| FWER p-Value | 0.031 |
Table: GSEA Results Summary

  

Fig 1: Enrichment plot: HALLMARK\_COMPLEMENT      
 Profile of the Running ES Score & Positions of GeneSet Members on the Rank Ordered List

  

| PROBE | DESCRIPTION (from dataset) | GENE SYMBOL | GENE\_TITLE | RANK IN GENE LIST | RANK METRIC SCORE | RUNNING ES | CORE ENRICHMENT || 1 | KCNIP2 | na |  |  | 59 | 3.598 | 0.0323 | No |
| 2 | MAFF | na |  |  | 475 | 2.376 | 0.0338 | No |
| 3 | COL4A2 | na |  |  | 1514 | 1.570 | -0.0052 | No |
| 4 | EHD1 | na |  |  | 1687 | 1.495 | 0.0005 | No |
| 5 | ERAP2 | na |  |  | 1876 | 1.416 | 0.0045 | No |
| 6 | PCLO | na |  |  | 2513 | 1.168 | -0.0174 | No |
| 7 | SH2B3 | na |  |  | 2717 | 1.112 | -0.0171 | No |
| 8 | BRPF3 | na |  |  | 2743 | 1.105 | -0.0075 | No |
| 9 | KCNIP3 | na |  |  | 2851 | 1.071 | -0.0026 | No |
| 10 | IRF7 | na |  |  | 3003 | 1.036 | -0.0004 | No |
| 11 | RAF1 | na |  |  | 3157 | 0.999 | 0.0014 | No |
| 12 | NOTCH4 | na |  |  | 3162 | 0.998 | 0.0110 | No |
| 13 | SIRT6 | na |  |  | 3243 | 0.978 | 0.0165 | No |
| 14 | PCSK9 | na |  |  | 3627 | 0.886 | 0.0051 | No |
| 15 | RASGRP1 | na |  |  | 3920 | 0.828 | -0.0021 | No |
| 16 | ZFPM2 | na |  |  | 4028 | 0.806 | 0.0002 | No |
| 17 | MMP15 | na |  |  | 4121 | 0.786 | 0.0031 | No |
| 18 | GP1BA | na |  |  | 4183 | 0.773 | 0.0075 | No |
| 19 | PIK3CG | na |  |  | 4509 | 0.713 | -0.0026 | No |
| 20 | PRDM4 | na |  |  | 4971 | 0.633 | -0.0205 | No |
| 21 | SERPINB2 | na |  |  | 4996 | 0.627 | -0.0156 | No |
| 22 | FYN | na |  |  | 5004 | 0.625 | -0.0098 | No |
| 23 | GATA3 | na |  |  | 5553 | 0.545 | -0.0332 | No |
| 24 | IRF1 | na |  |  | 5648 | 0.533 | -0.0329 | No |
| 25 | TMPRSS6 | na |  |  | 5664 | 0.530 | -0.0285 | No |
| 26 | STX4 | na |  |  | 5734 | 0.521 | -0.0270 | No |
| 27 | DGKH | na |  |  | 5742 | 0.521 | -0.0223 | No |
| 28 | SRC | na |  |  | 6020 | 0.482 | -0.0321 | No |
| 29 | LRP1 | na |  |  | 6291 | 0.446 | -0.0418 | No |
| 30 | CASP9 | na |  |  | 6333 | 0.439 | -0.0397 | No |
| 31 | CEBPB | na |  |  | 6839 | 0.379 | -0.0625 | No |
| 32 | TNFAIP3 | na |  |  | 6980 | 0.364 | -0.0662 | No |
| 33 | IL6 | na |  |  | 7244 | 0.333 | -0.0768 | No |
| 34 | DYRK2 | na |  |  | 7450 | 0.313 | -0.0844 | No |
| 35 | HSPA1A | na |  |  | 7497 | 0.308 | -0.0838 | No |
| 36 | C3 | na |  |  | 7510 | 0.306 | -0.0815 | No |
| 37 | JAK2 | na |  |  | 7561 | 0.298 | -0.0811 | No |
| 38 | CDK5R1 | na |  |  | 7644 | 0.286 | -0.0826 | No |
| 39 | RCE1 | na |  |  | 7763 | 0.278 | -0.0861 | No |
| 40 | CXCL1 | na |  |  | 7814 | 0.270 | -0.0861 | No |
| 41 | FDX1 | na |  |  | 7920 | 0.259 | -0.0890 | No |
| 42 | PIM1 | na |  |  | 8219 | 0.229 | -0.1024 | No |
| 43 | CP | na |  |  | 8317 | 0.218 | -0.1054 | No |
| 44 | C1S | na |  |  | 8670 | 0.184 | -0.1220 | No |
| 45 | DOCK4 | na |  |  | 9066 | 0.145 | -0.1413 | No |
| 46 | F3 | na |  |  | 9090 | 0.142 | -0.1411 | No |
| 47 | CBLB | na |  |  | 9113 | 0.138 | -0.1409 | No |
| 48 | KYNU | na |  |  | 9128 | 0.137 | -0.1403 | No |
| 49 | ME1 | na |  |  | 9632 | 0.084 | -0.1659 | No |
| 50 | C2 | na |  |  | 9829 | 0.060 | -0.1756 | No |
| 51 | CTSH | na |  |  | 9951 | 0.046 | -0.1815 | No |
| 52 | APOBEC3F | na |  |  | 9961 | 0.044 | -0.1815 | No |
| 53 | PRKCD | na |  |  | 10177 | 0.018 | -0.1926 | No |
| 54 | TIMP2 | na |  |  | 10178 | 0.018 | -0.1925 | No |
| 55 | CFB | na |  |  | 10199 | 0.015 | -0.1934 | No |
| 56 | CASP1 | na |  |  | 10302 | 0.015 | -0.1986 | No |
| 57 | GNB2 | na |  |  | 10378 | 0.009 | -0.2024 | No |
| 58 | AKAP10 | na |  |  | 10468 | -0.001 | -0.2071 | No |
| 59 | DOCK9 | na |  |  | 10669 | -0.026 | -0.2173 | No |
| 60 | APOBEC3G | na |  |  | 10765 | -0.034 | -0.2220 | No |
| 61 | DUSP5 | na |  |  | 10948 | -0.059 | -0.2309 | No |
| 62 | CASP10 | na |  |  | 10970 | -0.062 | -0.2314 | No |
| 63 | PRSS36 | na |  |  | 11231 | -0.087 | -0.2442 | No |
| 64 | RNF4 | na |  |  | 11420 | -0.104 | -0.2531 | No |
| 65 | PLAUR | na |  |  | 11509 | -0.112 | -0.2566 | No |
| 66 | PPP4C | na |  |  | 11656 | -0.132 | -0.2629 | No |
| 67 | CTSS | na |  |  | 11932 | -0.167 | -0.2757 | No |
| 68 | C1R | na |  |  | 12054 | -0.180 | -0.2803 | No |
| 69 | PPP2CB | na |  |  | 12099 | -0.185 | -0.2808 | No |
| 70 | APOC1 | na |  |  | 12152 | -0.193 | -0.2816 | No |
| 71 | IRF2 | na |  |  | 12188 | -0.198 | -0.2815 | No |
| 72 | CTSD | na |  |  | 12285 | -0.211 | -0.2845 | No |
| 73 | F8 | na |  |  | 12313 | -0.216 | -0.2838 | No |
| 74 | DPP4 | na |  |  | 12540 | -0.229 | -0.2934 | No |
| 75 | C4BPB | na |  |  | 12944 | -0.277 | -0.3118 | No |
| 76 | CFH | na |  |  | 12948 | -0.278 | -0.3092 | No |
| 77 | RHOG | na |  |  | 13032 | -0.288 | -0.3107 | No |
| 78 | CALM3 | na |  |  | 13042 | -0.289 | -0.3084 | No |
| 79 | USP16 | na |  |  | 13089 | -0.297 | -0.3079 | No |
| 80 | XPNPEP1 | na |  |  | 13136 | -0.304 | -0.3073 | No |
| 81 | PDP1 | na |  |  | 13308 | -0.327 | -0.3130 | No |
| 82 | PDGFB | na |  |  | 13460 | -0.347 | -0.3176 | No |
| 83 | DOCK10 | na |  |  | 13485 | -0.351 | -0.3154 | No |
| 84 | GNAI2 | na |  |  | 13525 | -0.357 | -0.3139 | No |
| 85 | LYN | na |  |  | 13668 | -0.380 | -0.3176 | No |
| 86 | HSPA5 | na |  |  | 13730 | -0.393 | -0.3170 | No |
| 87 | ANG | na |  |  | 13761 | -0.397 | -0.3146 | No |
| 88 | PLA2G4A | na |  |  | 13844 | -0.411 | -0.3149 | No |
| 89 | PLAT | na |  |  | 13867 | -0.414 | -0.3120 | No |
| 90 | PLG | na |  |  | 14094 | -0.429 | -0.3196 | No |
| 91 | SERPINA1 | na |  |  | 14126 | -0.430 | -0.3170 | No |
| 92 | GNB4 | na |  |  | 14403 | -0.477 | -0.3268 | No |
| 93 | PSEN1 | na |  |  | 14410 | -0.478 | -0.3224 | No |
| 94 | CLU | na |  |  | 14511 | -0.492 | -0.3228 | No |
| 95 | TIMP1 | na |  |  | 14560 | -0.499 | -0.3205 | No |
| 96 | LGMN | na |  |  | 15311 | -0.619 | -0.3537 | Yes |
| 97 | RABIF | na |  |  | 15405 | -0.635 | -0.3524 | Yes |
| 98 | CDA | na |  |  | 15484 | -0.647 | -0.3501 | Yes |
| 99 | LAP3 | na |  |  | 15631 | -0.674 | -0.3511 | Yes |
| 100 | PSMB9 | na |  |  | 15703 | -0.682 | -0.3482 | Yes |
| 101 | GRB2 | na |  |  | 15845 | -0.713 | -0.3485 | Yes |
| 102 | ZEB1 | na |  |  | 15994 | -0.743 | -0.3490 | Yes |
| 103 | GPD2 | na |  |  | 16014 | -0.750 | -0.3426 | Yes |
| 104 | USP8 | na |  |  | 16036 | -0.755 | -0.3363 | Yes |
| 105 | CTSB | na |  |  | 16096 | -0.769 | -0.3319 | Yes |
| 106 | PHEX | na |  |  | 16251 | -0.799 | -0.3321 | Yes |
| 107 | GCA | na |  |  | 16288 | -0.808 | -0.3260 | Yes |
| 108 | CASP4 | na |  |  | 16359 | -0.824 | -0.3216 | Yes |
| 109 | PFN1 | na |  |  | 16380 | -0.828 | -0.3145 | Yes |
| 110 | ADAM9 | na |  |  | 16444 | -0.841 | -0.3096 | Yes |
| 111 | TFPI2 | na |  |  | 16544 | -0.865 | -0.3062 | Yes |
| 112 | PRSS3 | na |  |  | 16638 | -0.883 | -0.3024 | Yes |
| 113 | GMFB | na |  |  | 16695 | -0.897 | -0.2966 | Yes |
| 114 | GNG2 | na |  |  | 16776 | -0.920 | -0.2917 | Yes |
| 115 | CSRP1 | na |  |  | 16883 | -0.950 | -0.2879 | Yes |
| 116 | KIF2A | na |  |  | 17054 | -1.001 | -0.2870 | Yes |
| 117 | VCPIP1 | na |  |  | 17141 | -1.028 | -0.2814 | Yes |
| 118 | PIK3CA | na |  |  | 17158 | -1.034 | -0.2721 | Yes |
| 119 | CD46 | na |  |  | 17237 | -1.060 | -0.2658 | Yes |
| 120 | CALM1 | na |  |  | 17246 | -1.065 | -0.2557 | Yes |
| 121 | PLSCR1 | na |  |  | 17416 | -1.120 | -0.2536 | Yes |
| 122 | PREP | na |  |  | 17813 | -1.292 | -0.2617 | Yes |
| 123 | CTSC | na |  |  | 17837 | -1.302 | -0.2501 | Yes |
| 124 | USP15 | na |  |  | 17862 | -1.315 | -0.2384 | Yes |
| 125 | GNAI3 | na |  |  | 17910 | -1.337 | -0.2278 | Yes |
| 126 | CASP7 | na |  |  | 18210 | -1.506 | -0.2286 | Yes |
| 127 | USP14 | na |  |  | 18211 | -1.507 | -0.2138 | Yes |
| 128 | S100A13 | na |  |  | 18233 | -1.519 | -0.2000 | Yes |
| 129 | DUSP6 | na |  |  | 18261 | -1.536 | -0.1863 | Yes |
| 130 | CTSO | na |  |  | 18318 | -1.582 | -0.1737 | Yes |
| 131 | ATOX1 | na |  |  | 18354 | -1.605 | -0.1598 | Yes |
| 132 | CD55 | na |  |  | 18406 | -1.643 | -0.1463 | Yes |
| 133 | PRCP | na |  |  | 18433 | -1.661 | -0.1313 | Yes |
| 134 | ANXA5 | na |  |  | 18489 | -1.699 | -0.1175 | Yes |
| 135 | LTA4H | na |  |  | 18589 | -1.771 | -0.1053 | Yes |
| 136 | CASP3 | na |  |  | 18683 | -1.879 | -0.0917 | Yes |
| 137 | LIPA | na |  |  | 18943 | -2.249 | -0.0832 | Yes |
| 138 | CPM | na |  |  | 19015 | -2.396 | -0.0634 | Yes |
| 139 | LAMP2 | na |  |  | 19026 | -2.430 | -0.0400 | Yes |
| 140 | CD59 | na |  |  | 19031 | -2.444 | -0.0162 | Yes |
| 141 | LGALS3 | na |  |  | 19056 | -2.536 | 0.0075 | Yes |
Table: GSEA details [plain text format]

  

Fig 2: HALLMARK\_COMPLEMENT      
 Blue-Pink O' Gram in the Space of the Analyzed GeneSet

  

Fig 3: HALLMARK\_COMPLEMENT: Random ES distribution      
 Gene set null distribution of ES for **HALLMARK\_COMPLEMENT**
